# Well-Defined Shell-Sheddable Core-Crosslinked Micelles with pH and Oxidation Dual-Response for On-Demand Drug Delivery

**DOI:** 10.3390/polym15091990

**Published:** 2023-04-23

**Authors:** Xinfeng Cheng, Qiyang Li, Xiaomeng Sun, Yuxin Ma, Huanping Xie, Weiguang Kong, Xianchao Du, Zhenghui Zhang, Dongfang Qiu, Yong Jin

**Affiliations:** 1 College of Chemistry and Pharmaceutical Engineering, Nanyang Normal University, Nanyang 473061, China; 2 Key Laboratory of Leather Chemistry and Engineering, Ministry of Education, National Engineering Research Center of Clean Technology in Leather Industry, Sichuan University, Chengdu 610065, China

**Keywords:** shell-sheddable, core-crosslinking, pH-responsive, oxidation-responsive, drug delivery

## Abstract

Micellar-nanocarrier-based drug delivery systems possessing characteristics such as an excellent circulation stability, inhibited premature release and on-demand site-specific release are urgently needed for enhanced therapeutic efficacy. Therefore, a novel kind of shell-sheddable core-crosslinked polymeric micelles with pH and oxidation dual-triggered on-demand drug release behavior was facilely constructed. The multifunctional micelles were self-assembled from a carefully designed amphiphilic triblock PEGylated polyurethane (PEG-*acetal*-PUBr-*acetal*-PEG) employing an acid-labile acetal linker at the hydrophilic–hydrophobic interface and pendant reactive bromo-containing polyurethane (PU) as the hydrophobic block, followed by a post-crosslinking via oxidation-cleavable diselenide linkages. These well-defined micelles exhibited an enhanced structural stability against dilution, achieved through the incorporation of diselenide crosslinkers. As expected, they were found to possess dual pH- and oxidation-responsive dissociation behaviors when exposure to acid pH (~5.0) and 50 mM H_2_O_2_ conditions, as evidenced using dynamic light-scattering (DLS) and atomic force microscopy (AFM) analyses. An in vitro drug release investigation showed that the drug indomethacin (IND) could be efficiently encapsulated in the micelles, which demonstrated an inhibited premature release compared to the non-crosslinked ones. It is noteworthy that the resulting micelles could efficiently release entrapped drugs at a fast rate in response to either pH or oxidation stimuli. Moreover, the release could be significantly accelerated in the presence of both acid pH and oxidation conditions, relative to a single stimulus, owing to the synergetic degradation of micelles through pH-induced dePEGylation and oxidation-triggered decrosslinking processes. The proposed shell-sheddable core-crosslinked micelles with a pH and oxidation dual-response could be potential candidates as drug carriers for on-demand drug delivery.

## 1. Introduction

Nanomicelles with hydrophobic cores and hydrophilic shells, assembled with amphiphilic polymers, have been found to be a kind of ideal nanocarriers for gene and drug delivery [1,2,3]. In recent decades, polymeric micelles with stimuli-sensitivity have emerged as promising smart nanocarriers due to their on-demand dissociation and triggered release of entrapped drugs at a desired site in response to intracellular or external environmental changes, such as pH, redox, temperature, light, magnetic field and ultrasound [4,5,6,7]. However, there is still a practical challenge that drug-loaded polymeric micelles are easily diluted or severely sheared in blood circulation after being intravenously injected, leading to a dissociation and premature release of the loaded drugs at undesired sites, resulting in insufficient drug availability and even toxic side-effects on normal tissues [8,9]. In order to solve this restrictive problem, crosslinking is one of the most effective strategies applied to stabilize the self-assembled micelles. Through core, shell or core–shell interface crosslinking, the circulation stability of the drug-loaded polymeric micelles was effectively improved so as to attain an efficient therapeutic efficacy [10,11,12,13].

However, an excessive stability of the crosslinked micelles was found to be unfavorable for the drug delivery at the target sites, potentially leading to a slow and incomplete release of the encapsulated drugs. Notably, stimuli-responsive crosslinking provided an effective approach to solve this restriction. By introducing stimuli-cleavable crosslinkers into the micelles, smart crosslinked micellar nanocarriers with a reversible crosslinking property were achieved, which possessed not only a prolonged circulation stability in the extracellular matrix, but also the selective release of encapsulated drugs at the desired sites triggered using appropriate intracellular or extracorporeal stimuli (pH, temperature, photo, redox, etc.)-induced micelle dissociation [14,15,16,17]. Today, redox-responsive disulfide-crosslinked polymeric micelles have been extensively studied due to their desirable crosslinking holding or decrosslinking behaviors that exist in blood circulation or the cytoplasm of tumor cells with different orders of magnitude for redox potential [18,19]. As an analogue of sulfide, selenium, with its lower electronegativity, was developed to construct stimuli-responsive polymers owning higher redox-sensitivity, as compared to sulfide. Therefore, a series of diselenide-containing polymers were designed and investigated for biomedical applications due to the diselenide bond being easier to cleave through redox potential, i.e., glutathione (GSH) and reactive oxygen species (ROS) [20,21,22]. Despite the promising features of the diselenide bond and there being some reports focusing on diselenide-crosslinked micelles [23,24,25,26], there is still a demand for the development of more advanced micelles crosslinked using a redox-sensitive diselenide bond.

The PEGylation of micelles was developed into another useful approach to improve the circulation stability of the self-assembled polymeric micelles [27,28]. As for PEGylated micelles, the formation of hydrophilic PEG shells can protect the micelles from interactions with proteins in the bloodstream or uptake by macrophages [29]. Nevertheless, PEGylation is a double-edged sword, bringing about negative influences as well. The hydrophilic PEG shells also serve as an effective barrier to the outward diffusion of the entrapped drugs, resulting in a slow and sustained drug release at the target site, as well as an inefficient therapeutic efficacy [30,31]. To overcome this limitation, the stimuli-responsive shedding of PEG shells was found to be an efficient method, which is similar to the aforementioned stimuli-responsive crosslinking method. For instance, shell-sheddable micellar nanocarriers can be prepared by using amphiphilic copolymers with a disulfide, imine or orthoester bond as the linkers between the hydrophilic block and the hydrophobic block, as a result exhibiting a faster on-demand intracellular drug release and higher therapeutic efficacy than their nonsheddable counterpart [32,33,34]. Despite many kinds of PEG-based shell-shedding micellar nanocarriers having been designed and prepared, the development of advanced shell-shedding micelles-based nanocarriers integrated with two or more endogenous stimuli-responsive labile linkages for a dual or multistimuli response is still challenging. Moreover, it can be expected that the dual or multistimuli responsiveness characteristics may endow the micelles with enhanced biodegradability and provide more choices for efficient drug release when there is exposure to a complex tumor microenvironment with dual or more complicated and changeable stimuli signals.

Recently, acetal linkages have been extensively explored as effective acid-labile linkages for pH-triggered on-demand drug delivery systems [35]. Among them, cyclic benzylidene acetals containing amphiphilic polymeric micelles have been the most widely explored as pH-responsive nanodrug delivery systems due to their quick cleavage within the physiologically accessible pH range of 4.0–6.5 [36]. Moreover, because of the strong π–π interactions between the aromatic rings, this kind of cyclic benzylidene acetal bearing amphiphilic polymers may not only self-assemble into micelles at a lower critical micellar concentration (CMC), but also efficiently encapsulate common hydrophobic drugs with aromatic or cyclic ring structures [37]. Therefore, the incorporation of pH-breakable acetal linkages, especially cyclic benzylidene acetals, into polymer micelles as pH-sensitive intracellular nanocarriers has gained widespread attention [35,38].

Herein, in the present study, diselenide core-crosslinked and shell-sheddable amphiphilic polyurethane (PU) micelles with cyclic acetal linkages at block junctions were developed and investigated, aimed at improving their circulation stability and on-demand dual pH- and oxidation-triggered drug release properties. The amphiphilic triblock copolymer PEG-*acetal*-PUBr-*acetal*-PEG consisting of a pH-sensitive cyclic acetal linker at the hydrophilic–hydrophobic interface and dangling reactive Br groups in the PU block were facilely designed. The amphiphilic ABA-type copolymer could self-assemble into micelles, whose core could be further crosslinked with diselenide linkages using sodium diselenide additives. The dilution-resistant stability of the resultant crosslinked micelles was examined with dynamic light-scattering (DLS) and atomic force microscopy (AFM). The disassembly behavior of the corresponding micelles was explored in detail upon exposure to acid pH and oxidative conditions. Moreover, by choosing indomethacin (IND) as a model drug, the drug loading and on-demand drug release behavior triggered through the pH and oxidation dual-response was also investigated.

## 2. Materials and Methods

### 2.1. Materials

Isophorone diisocyanate (IPDI, 99%), dibutyltin dilaurate (DBTDL, 95%), p-hydroxybenzaldehyde (p-HBD, 98%) and p-toluenesulfonic acid (p-TSA, 98%) were purchased from Shanghai Aladdin reagent Co., LTD (Shanghai, China). Ymer^TM^ N120, a commercially available α-methoxy-ω-diol poly(ethylene glycol) (Mn = 1000), was purchased from Perstorp special chemicals (Malmö, Sweden) and vacuum-dried at 80 °C for 12 h before use. 2,2-Bis(bromomethyl)propane-1,3-diol (BMP, 98%) and indomethacin (IND, >98%) were purchased from TCI chemicals (Shanghai, China). Sodium diselenide (Na_2_Se_2_) was synthesized according to the literature [39]. Other reagents of analytical grade were purchased from Tianjin Kemiou Chemical Reagent Co., Ltd. (Tianjin, China), and used as received.

### 2.2. Instrumentation

The ^1^H-NMR spectrum was recorded on a JEOL JNM-ECA 300 (300 MHz) spectrometer (JEOL, Tokyo, Japan). The gel permeation chromatography (GPC) measurement was performed on an HLC-8320 GPC (Tosoh, Tokyo, Japan) using polystyrene as the standard and THF as the eluent. Fourier transform infrared (FT-IR) spectra were recorded on a ThermoFisher Nicolet 6700 spectrophotometer (Thermo Fisher Scientific, Waltham, MA, USA) in KBr pellets. Raman spectra of the samples were recorded with a LabRam spectrometer (Horiba, Kyoto, Japan) using a He–Ne laser with an emission wavelength of 633 nm. A Hitachi U-2010 spectrophotometer (Hitachi, Tokyo, Japan) was used to record the transmittance and absorbance of the samples. Atomic force microscopy (AFM) images were obtained in the tapping mode with a Shimadzu Scanning Probe Microscope (SPM-9600, Shimadzu, Kyoto, Japan) operated in air. Samples for the AFM analysis were prepared through spin-casting onto a mica surface from the solutions of polymer nanoparticles. The sizes and distributions of polyurethane nanoparticles were measured with a Zetasizer Nano ZS dynamic light-scattering (DLS) instrument (Malvern Instruments, Malvern, UK) at 25 °C at an angle of 90°.

### 2.3. Synthesis of PEG-Acetal-PUBr-Acetal-PEG Block Copolymers

**Scheme 1:**
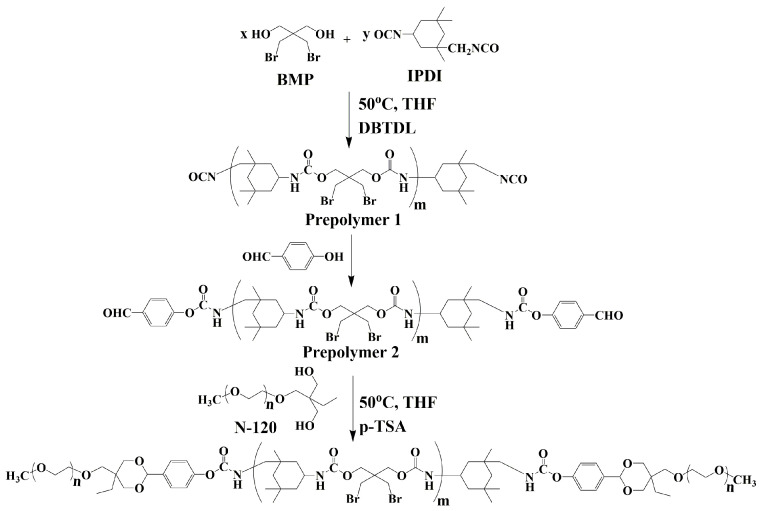
The synthetic route of PEG-*acetal*-PUBr-*acetal*-PEG block copolymers.

PEG-*acetal*-PUBr-*acetal*-PEG was synthesized through two sequential reactions, as displayed in Scheme 1. Firstly, the polycondensation reactions of IPDI (6.6 mmol, 1.47 g) and BMP (6 mmol, 1.57 g) were processed at 50 °C using DBTDL as the catalyst in 25 mL of a dried tetrahydrofuran (THF) solution under a N_2_ atmosphere. Upon stirring for 2 h, p-HBD (0.37 mmol, 0.045 g) and a triethylamine analyst were added and stirred at 50 °C for 24 h. Then, the prepolymer PUBr with active aldehyde terminals was obtained. Afterwards, the acetalization reaction of benzaldehyde and diol (Ymer^TM^ N120, Malmö, Sweden) was performed. In brief, Ymer^TM^ N120 (0.40 mmol, 0.40 g), a catalytic amount of p-TSA and 5Å molecular sieves were added to the above system, and the reaction was maintained at 50 °C for another 24 h. Then, the resulting reaction mixture was precipitated in diethyl ether and further purified through dissolution in THF and precipitation in diethyl ether two more times. After vacuum drying at room temperature overnight, the final copolymer PEG-*acetal*-PUBr-*acetal*-PEG with a yield of 78% was obtained.

### 2.4. Preparation of Non-Crosslinked PEG-Acetal-PUBr-Acetal-PEG Micelles and Core-Crosslinked PEG-Acetal-PUSe-Acetal-PEG Micelles

The non-crosslinked PEG-*acetal*-PUBr-*acetal*-PEG micelles were prepared with the solvent-exchange method. As customary, the triblock copolymer PEG-*acetal*-PUBr-*acetal*-PEG (20 mg) was dissolved in 2 mL of THF. Under stirring, the obtained solution was dropwise added into 40 mL of distilled water and incubated at room temperature overnight to achieve equilibrium. Next, the solution was sealed inn a dialysis bag (cutoff 3500 Da) and dialyzed against distilled water (~1000 mL) for 24 h. Then, the non-crosslinked PEG-*acetal*-PUBr-*acetal*-PEG micelles were obtained and filtrated via a 0.45 μm filter prior to use.

The diselenide core-crosslinked micelles were prepared via the post-crosslinking method using Na_2_Se_2_ as the crosslinker. The Na_2_Se_2_ solution (~0.2 M) was prepared via the same procedure described in our previous work [40], and then dropped into the as-prepared PEG-*acetal*-PUBr-*acetal*-PEG micelles. The above mixture solution was stirred at 37 °C for 12 h, followed by dialysis against distilled water for 24 h. Then, the core-crosslinked micelles named PEG-*acetal*-PUBr-*acetal*-PEG were obtained and filtrated through a 0.45 μm filter for further use. The micellar parameters, including the particle size, distribution and morphology of the non-crosslinked and core-crosslinked micelles, were recorded using dynamic light scattering (DLS, Malvern Instruments, Malvern, UK) and atomic force microscopy (AFM, SPM-9600, Shimadzu, Kyoto, Japan).

### 2.5. Structural Stability of Micelles

To test the structural stability of the crosslinked micelles, DMF, a good solvent for the dissolution of the synthesized copolymers, was chosen and added into the micelle solutions to determine their structural stability. As customary, different volumes (V_DMF_/V_H2O_ = 0/100 ~ 50/50) of DMF were injected into the as-prepared non-crosslinked or core-crosslinked micelles. Meanwhile, the scattering light intensities of the diluted micelles were recorded through the DLS analysis to evaluate their structural stability. In addition, the aqueous micelle solutions were freeze-dried and redispersed in the DMF solvent to further test their structural stability. The corresponding properties of the micelles before and after the dilution treatment, such as scattering light intensity, particle size, distribution and morphology, were characterized using DLS and AFM.

### 2.6. pH- and Oxidation-Induced Disassembly of Micelles

To investigate the pH- and oxidation-triggered disassembly behaviors of the micelles, 5 mL of the above prepared diselenide-crosslinked micelles was added into a dialysis bag (cutoff 3500 Da) and placed in a pH 5.0 and pH 7.4 PBS solution with 50 mM H_2_O_2_ or without, respectively. Upon exposure to the above single or dual stimuli at room temperature for 24 h, the progressive changes in particle size, distribution and morphology transformation were recorded through DLS and AFM.

### 2.7. Drug Loading and Release Studies

The hydrophobic indomethacin (IND) was selected as a model drug, with the IND-loaded micelles prepared using a coprecipitation method. As customary, a certain amount of IND (20 mg) and copolymer PEG-acetal-PUBr-acetal-PEG (150 mg) were added into 5 mL of THF, followed by a sonication treatment (10 min) and continuous stirring overnight. After the drop-wise addition of the THF solution into 100 mL of distilled water and continuous stirring for 12 h, the freshly synthesized Na_2_Se_2_ solution (~0.2 M) was added into the mixture solution and incubated at 50 °C under stirring overnight. Afterwards, the above solution system was dropped into a dialysis bag and dialyzed against distilled water at room temperature to remove the residual Na_2_Se_2_, THF and unentrapped IND. After 48 h, the IND-loaded diselenide-crosslinked micelles were obtained, which were then further filtrated using a 0.45 μm filter membrane before use. The amount of IND entrapped in the micelles was determined using a UV/Vis spectrometer (Hitachi U-2010, Tokyo, Japan) through a comparison with a standard curve at 320 nm. The loading parameters, such as the drug loading content (DLC) and drug loading efficiency (DLE), were determined based on the formula below:

DLC = (weight of loaded IND / weight of IND loaded micelles) × 100%(1)

DLE = (weight of loaded IND / weight of IND in feed) × 100%(2)

The stimuli-triggered drug release behavior of the micelles was studied in response to acid pH and/or oxidative stress. In total, 5 mL of the IND-loaded diselenide-crosslinked micelles was transferred into a dialysis bag (cutoff 3500 Da) and put into different release media, such as pH 7.4 PBS, pH 6.5 PBS, pH 5.0 PBS, pH 7.4 PBS with 50 mM H_2_O_2_, pH 6.5 PBS with 50 mM H_2_O_2_ and pH 5.0 PBS with 50 mM H_2_O_2_. Upon mild shaking (120 rpm) at 37 °C, a certain volume (3 mL) of the outer release medium was taken at appropriate periods and analyzed with a UV/Vis spectrometer, which was in time replenished with the same volume of fresh medium. The released amount of payloads was determined using the recorded absorbance at 320 nm based on the standard curve. All release experiments were conducted in triplicate.

## 3. Results and Discussion

### 3.1. Synthesis and Characterization of PEG-Acetal-PUBr-Acetal-PEG Block Copolymers

To develop a feasible and efficient nanoplatform as a potential carrier for stimuli-triggered on-demand drug delivery, the novel well-defined amphiphilic copolymer PEG-*acetal*-PUBr-*acetal*-PEG, with a sheddable shell and reversible crosslinkable core, was facilely synthesized. As illustrated in Scheme 1, the triblock copolymer PEG-*acetal*-PUBr-*acetal*-PEG could be readily synthesized via two sequential polycondensation and acetalization reactions. First, a benzaldehyde-terminated polyurethane prepolymer PUBr bearing dangling bromo groups was synthesized through a polycondensation reaction of IPDI with BMP and subsequent end-capping using p-HBD. Then, an acetal linker was introduced to the hydrophobic–hydrophilic interface via the acetalization reaction of the active benzaldehyde terminals with Ymer^TM^ N120, an adjacent diol containing a PEG derivative.

**Figure 1:**
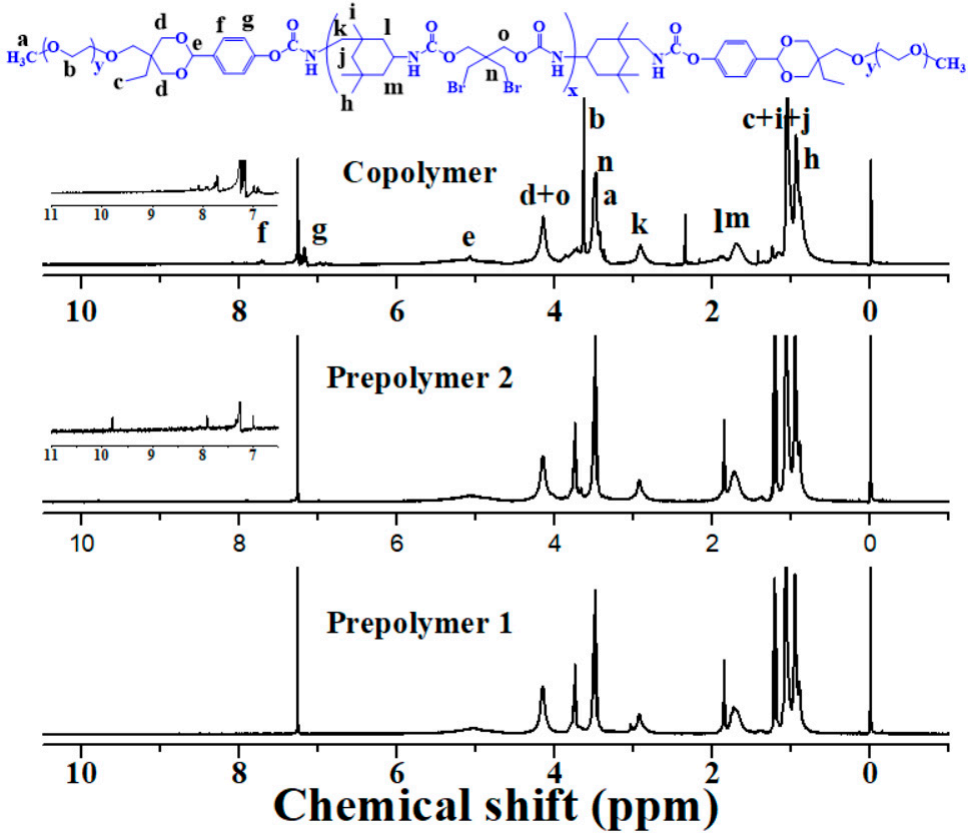
The ^1^H NMR spectra of the PEG-*acetal*-PUBr-*acetal*-PEG block copolymer and its prepolymers 1 and 2.

The chemical structure and composition of the functional copolymer PEG-*acetal*-PUBr-*acetal*-PEG were examined through ^1^H NMR, FT-IR and GPC analyses. The ^1^H NMR spectra of the copolymer PEG-*acetal*-PUBr-*acetal*-PEG, as well as its prepolymers one and two, were displayed in Figure 1. As shown in Figure 1, their ^1^H NMR spectra displayed that the chemical shifts ranging from 0.83 to 2.91 ppm originated from the IPDI units; the shifts at 3.43 and 4.15 ppm were, respectively, assigned to the –CH_2_Br and –O–CH_2_–C–CH_2_–O– of BMP units. Prepolymer two exhibited characteristic signals at 6.98/7.91 and 9.80 belonging to the phenyl ring and the –CHO protons of p-HBD, whereas it should be noted that the signal at approximately 9.8 ppm disappeared, and a characteristic signal belonging to cyclic acetal located at 5.1 ppm emerged for the copolymer, informing on the successful acetalization reaction between the benzaldehyde and diol of N120. Meanwhile, there existed a strong peak belonging to the –CH_2_CH_2_O– of Ymer^TM^ N120 at approximately 3.50 ppm for the final copolymer.

**Figure 2:**
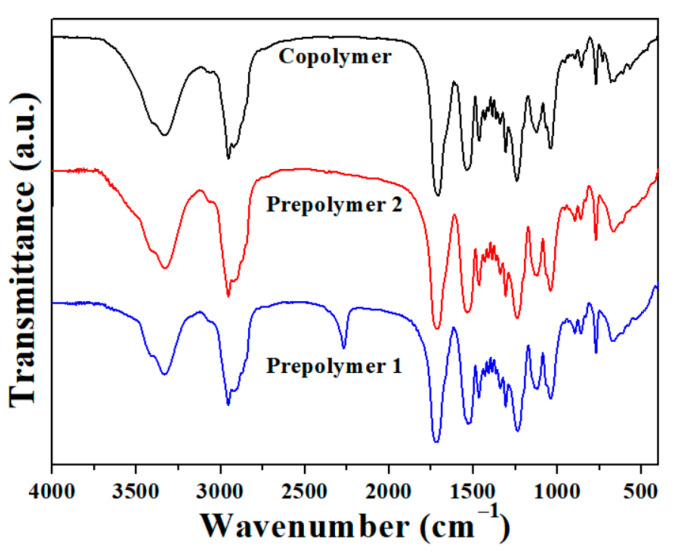
The FT-IR spectra of the PEG-*acetal*-PUBr-*acetal*-PEG block copolymer and its prepolymers 1 and 2.

**Figure 3:**
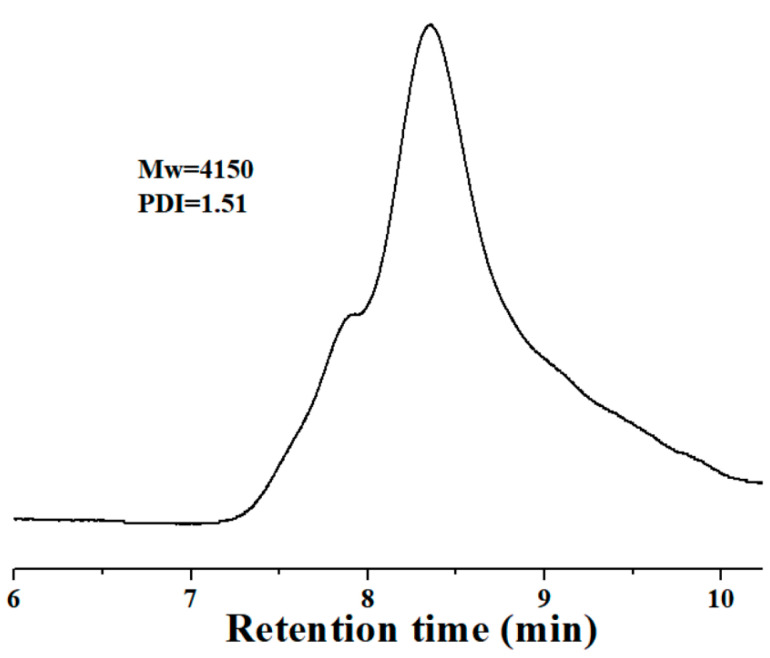
The GPC trace of the PEG-*acetal*-PUBr-*acetal*-PEG block copolymer.

The FT-IR spectra of the copolymer and its prepolymers one and two were also illustrated in Figure 2. The characteristic bands that appeared at 1708 and 1534 cm^−1^ belonged to the resultant urethane groups for all the prepolymers and the final copolymer. Additionally, the absorption peaks at 1307 cm^−1^ were attributed to the –CH_2_– (bromomethyl) wagging vibration of the BMP units. As for prepolymer one, a distinct absorption peak located at 2270 cm^−1^ was observed, which was ascribed to the stretching vibration of the characteristic -NCO groups. Additionally, it disappeared when adding p-HBD as the end-capping agent, indicating the complete reaction conversion of the remaining –NCO groups from the IPDI units to afford prepolymer two. From the spectrum of the final copolymer PEG-*acetal*-PUBr-*acetal*-PEG, the absorption peaks at 1118 cm^−1^ were ascribed to the C–O–C stretching vibration of the PEG (N120) segments, and the bands at 731 and 660 cm^−1^ were assigned to the vibration of the phenyl rings. A further analysis with GPC demonstrated that the copolymer had a molecular weight of 4150 (M_w_), with a polydispersity of 1.51 (Figure 3). According to the above analyses, a rationally designed triblock copolymer PEG-*acetal*-PUBr-*acetal*-PEG was successfully synthesized.

### 3.2. Preparation and Characterization of the Diselenide-Crosslinked Micelles

**Figure 4:**
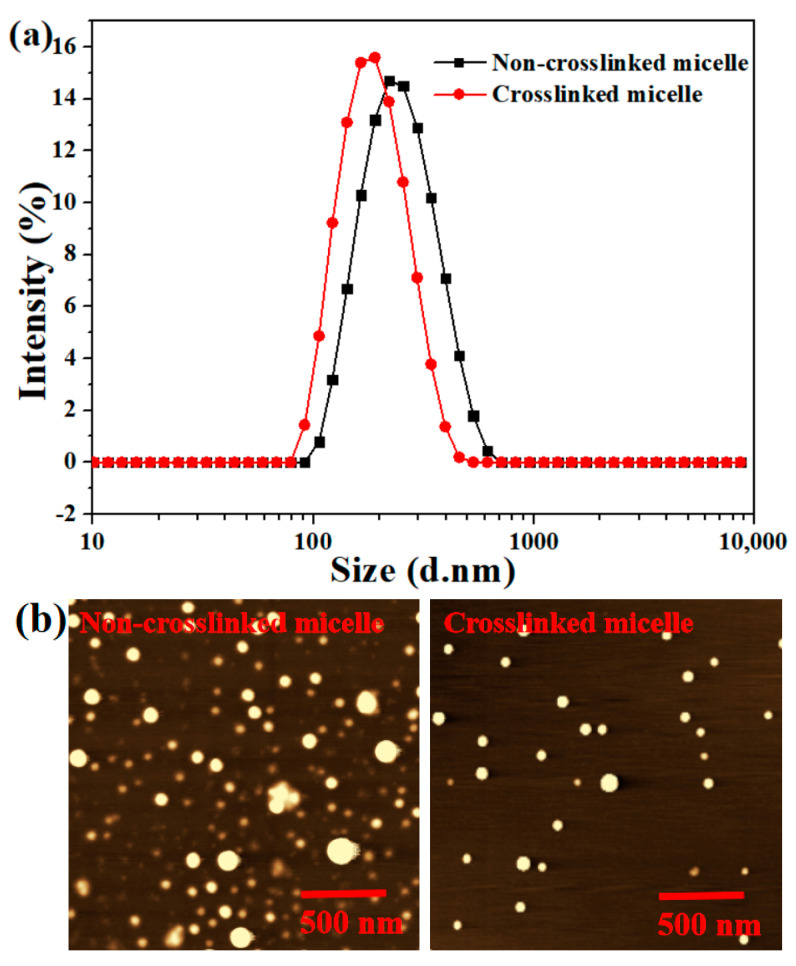
The DLS data (**a**) and AFM images (**b**) of micelles before and after crosslinking.

The diselenide-crosslinked micelles were prepared via two sequential steps using the micellization and post-crosslinking methods. Owing to the amphiphilic property of the designed triblock copolymers, PEG-*acetal*-PUBr-*acetal*-PEG copolymers tend to self-aggregate and form micellar nanoparticles in aqueous solutions. As illustrated in Scheme 1, the well-defined copolymer could self-assemble into micelles composed of hydrophobic PU cores and hydrophilic PEG shells. Herein, a widely reported solvent exchange method was adopted to prepare the PEG-*acetal*-PUBr-*acetal*-PEG micelles. The particle size and micromorphology of the self-assembled micelles were revealed using DLS and AFM. The DLS data showed that the hydrodynamic diameter (*D_h_*) of the as-prepared micelles was 234 nm, with a polydispersity index (PDI) of 0.155 (Figure 4). From the AFM image shown in Figure 4, it could be seen that the micelles presented a spherical structure with a diameter ranging from 130 to 200 nm.

The PEG-*acetal*-PUBr-*acetal*-PEG micelles were post-crosslinked through diselenide linkages using Na_2_Se_2_ as the crosslinker. The as-prepared micelles had the dangling Br groups in their hydrophobic domains, which were active to react with Na_2_Se_2_ through the nucleophilic substitution reaction in a general aqueous environment, yielding the diselenide-crosslinked PEG-*acetal*-PUBr-*acetal*-PEG micelles. Further DLS and AFM measurements displayed that the micelle aggregates were shown to be more uniform after crosslinking, whose diameters significantly decreased to 188 nm, along with a narrow distribution of PDI~0.132. This phenomenon resulted from the formation of a more compact micellar structure after crosslinking.

### 3.3. Stability of the Micelles

The stability of the micellar nanocarriers plays a pivotal role in maintaining their self-assembly structure during blood circulation. To test the structural stability of the crosslinked micelles, DMF, a well-known solvent for the dissolution of the synthesized amphiphilic copolymers, was chosen and added into the micelle solutions to determine their structural stability. The corresponding properties of the micelles before and after the dilution treatment, such as scattering light intensity, particle size, distribution and morphology, were characterized using DLS and AFM.

**Figure 5:**
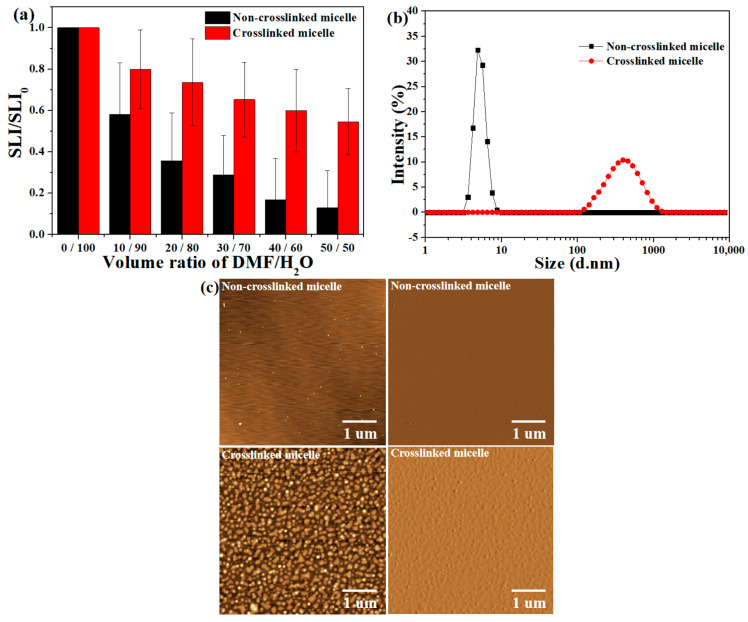
The scattering light intensity (SLI) variation (**a**), DLS data (**b**) and AFM images (**c**) (left: height image; right: phase image) of micelles against volume dilution before and after crosslinking.

**Figure 6:**
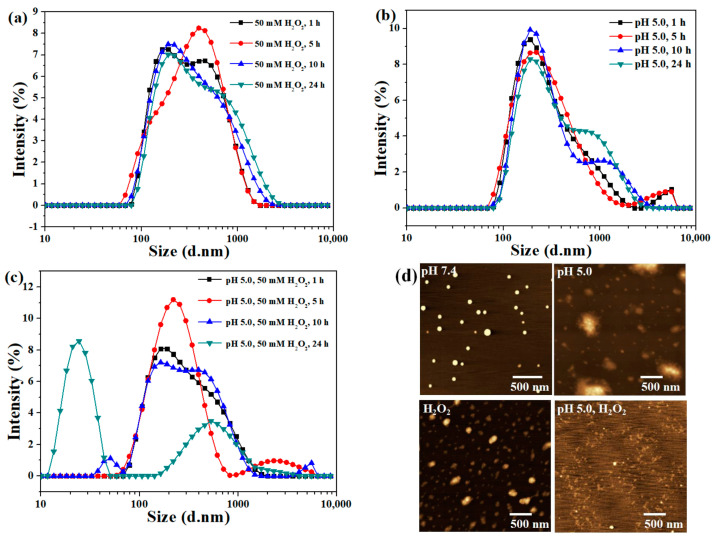
The DLS data (**a**–**c**) and AFM variation images (**d**) of micelles before and after crosslinking under acidic and/or oxidation stimuli.

By adding different volume ratios of the DMF solvent into the as-prepared non-crosslinked or core-crosslinked micelle aqueous solutions, the corresponding scattering light intensity (SLI) of the diluted micelles was recorded using the DLS analysis, as depicted in Figure 5. It was evident from the data that the SLI of the non-crosslinked PEG-*acetal*-PUBr-*acetal*-PEG micelles underwent a sharp decrease of more than 40% upon the addition of 10% percentage of DMF, which continuously decreased up to 13% upon further dilution. Comparably, the crosslinked PEG-*acetal*-PUBr-*acetal*-PEG micelles demonstrated a smaller decrease in SLI, along with a 55% retention after a 50% dilution of the DMF. These observations implied that the self-assembled micelles after crosslinking were stable enough to withstand harsh conditions. In addition, the micelle aqueous solutions were freeze-dried and redispersed in the DMF solvent to further test their structural stability against harsher environment. As was evidenced from the DLS and AFM results shown in Figure 6, there existed only a narrow distribution of approximately 5 nm and no assembles, but some unimers appeared on the mica surface for the non-crosslinked micelles, while, as a contrast, the diselenide-crosslinked micelles exhibited a larger size of approximately 380 nm, with a PDI of 0.379 in the DMF as compared to the micelles in water (188 nm). It was especially apparent from the AFM analysis that the freeze-dried crosslinked samples exhibited a swollen core–shell structure upon being dispersed in DMF. In view of the findings presented above, it could be concluded that these diselenide-crosslinked micelles could effectively hold their assembled structure against dilution in extreme environments compared to the non-crosslinked ones, which are highly needed as ideal micellar nanocarriers. Additionally, the crosslinked micelles in the aqueous solution also showed the long-term colloidal stability, whose size remained unchanged when exposed in a conventional environment throughout approximately 30 days.

### 3.4. The pH- and Oxidation-Responsive Behaviors of the Diselenide-Crosslinked Micelles

The stimuli responsiveness of the well-defined micelles in response to pH and/or oxidation stimuli was investigated through DLS and AFM measurements. As shown in Figure 6a, the crosslinked micelles reflected an obvious change in particle size under a continuous oxidation stimulus (50 mM H_2_O_2_). The particle size of the crosslinked micelles increased to 269 nm in the initial 1 h, and continued to grow to 504 nm after 24 h, accompanied by a distinct broadening of the PDI. This was attributed to the oxidation-induced cleavage of diselenides-based crosslinkages, yielding more hydrophilic selenic acid groups and, thus, giving rise to a more swollen structure. In addition, it was clear to see that the particle size and PDI also gradually increased, and some bigger assembles beyond 1000 nm formed at acidic conditions (pH 5.0), as depicted in Figure 6b. The possible reason is that the hydrophilic PEG shells of the micelles shed upon exposure to acidic circumstances via acetal bond cleavage [19], resulting in the accumulation of the exposed hydrophobic inner cores of micelles, thus, forming larger aggregate structures.

Furthermore, the dual-responsive behaviors in the presence of both acidic pH and oxidation conditions were also explored. As shown in Figure 6c, the particle size increased gradually from 188 nm to 315 nm in 1 h, and then reflected a marked bimodal distribution of approximately 300 and 1000 nm in 5 h, while some particles of smaller size of approximately 25 nm and bigger ones of approximately 1000 nm began to appear upon further treatment for 24 h, which was accompanied by a wide range of size distributions. In the meantime, a similar structural variation of the diselenides crosslinked micelles under single or dual stimuli was also observed in the AFM images, as reflected in Figure 6d. These observations indicated that the diselenide-crosslinked micelles could undergo a pH-induced PEG shell detachment and oxidation-triggered PU core decrosslinking behaviors, giving rise to the disassembly of micelles and the formation of more hydrophilic polymer fragments, and even their large aggregates.

### 3.5. In Vitro Stimuli-Responsive Drug Release of Drug-Loaded Core-Crosslinked Micelles

In order to evaluate the drug release characteristics of the core-crosslinked micelles, the hydrophobic indomethacin (IND) was selected and encapsulated in the micelles as a model drug. The drug loading parameters, such as the drug loading content (DLC) and drug loading efficiency (DLE), were determined to be 20.6% and 73.2%, respectively, which were comparable to other related micellar nanocarriers.

**Figure 7:**
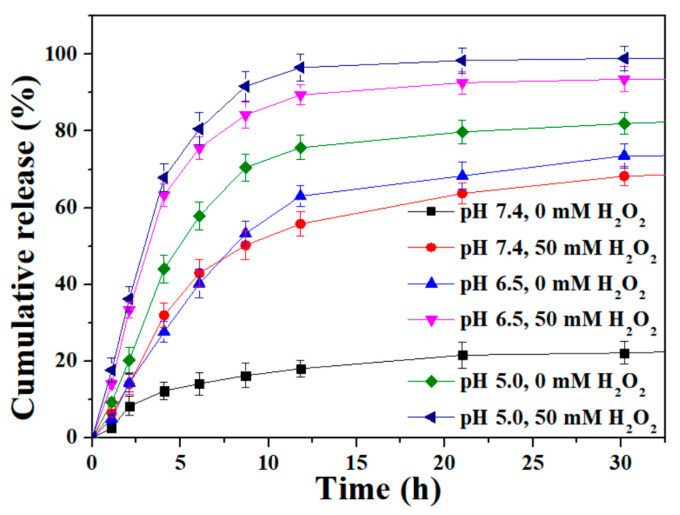
The cumulative release profiles from IND-loaded core-crosslinked micelles under different pH and/or oxidation environments.

The stimuli-responsive drug release behaviors of the micelles were examined under different pH and/or oxidative circumstances. The release experiments were carried out via the dialysis method using different release media to mimic the pH and oxidative stress levels in cytoplasmic microenvironments, such as pH 7.4, 6.5 or 5.0 PBS, with or without 50 mM H_2_O_2_ at approximately 37 °C, as shown in Figure 7. At pH 7.4, without H_2_O_2_, the IND-loaded core-crosslinked micelles exhibited a sustained release, with only approximately 14% drug leakage for 6 h and less than 22% release within 30 h. This relatively slow-release behavior could probably be attributed to the formation of an effective barrier that hindered drug diffusion through the efficient core-crosslinking method, while when decreasing the pH to acidic conditions (pH 6.5 or 5.0), a remarkable boost release manner was observed for the IND-loaded core-crosslinked micelles. There was approximately 63% of IND cargoes released within 12 h and a cumulative release up to 74% after 30 h for pH 6.5. When the pH value further decreased to 5.0, a faster release behavior was observed and approximately 75% of IND was released in 12 h, and 82% after 30 h. This revelation indicated that the acid pH-induced shell-shedding behavior that resulted from the acetal bond cleavage could destroy the shell barrier of the micellar nanocarriers, thus, accelerating the release of the hydrophobic IND drugs loaded in the inner core of the micelles.

When exposed to oxidation conditions (50 mM H_2_O_2_) at pH 7.4, the release rate of IND increased more quickly, with approximately 56% released within 12 h. After 30 h, the cumulative release of IND was up to 68% in this oxidative condition. The accelerated release in response to H_2_O_2_ should have contributed to the oxidation-triggered core-decrosslinking behavior derived from the diselenide bond breakage, which promoted the swelling performance of the micelles and facilitated the leakage of the hydrophobic IND cargoes. Moreover, the release profiles of the IND-loaded core-crosslinked micelles in the presence of dual pH and oxidation stimuli were also studied. As shown in Figure 7, it was remarkable to see a burst release of approximately 90% in 12 h and 94% after 30 h at pH 6.5 with 50 mM H_2_O_2_, demonstrating an enhanced drug bioavailability, which is highly on-demand for nanocarriers to deliver drugs at specific target sites. Furthermore, an intensified burst release occurred and an approximately complete release (95% in 12 h) was observed at pH 5.0 with 50 mM H_2_O_2_.

Owing to the synergy effect of the dual pH- and oxidation-triggered disassembly behavior of the shell-sheddable core-crosslinked micelles, they could efficiently release entrapped drugs at a much faster rate than at either acidic pH or oxidation conditions, which is desirable for ideal nanocarriers.

Furthermore, the Ritger and Peppas equation was used as an empirical model for the description of the probable dissolution and diffusion mechanism of the entrapped drugs [40,41], as shown below:

*M_t_* / *M_∞_* = *kt^n^* (for *M_t_* / *M_∞_* ≤ 0.6) (3)

where *M_t_* and *M_∞_* represent the cumulative release of the drug at time *t* and infinite time, respectively, *k* is the rate constant and *n* is the release exponent, revealing the release mechanism. For instance, if *n* = 0.5, the drug release mechanism would be Fickian diffusion. If *n* = 1, the model would be a zero-order release. If 0.5 < *n* < 1, the model would be an anomalous transport, and the structural relaxation would be comparable to diffusion. If *n* < 0.5, the release mechanism would belong to a pseudo-Fickian diffusion.

The kinetic model parameters obtained through curve fitting the in vitro drug release results using the Ritger and Peppas empirical equation were summarized in Table 1. At pH 7.4, medium, without H_2_O_2_, the corresponding *n* value was predicted to be 0.45, implying a pseudo-Fickian diffusion mechanism in this case, while in the presence of acidic and/or oxidation conditions, the n values increased from 0.62 (pH 6.5) and 0.66 (50 mM H_2_O_2_) under single stimulus, and then to approximately 0.79 (pH 6.5/5.0 and 50 mM H_2_O_2_) under the combined stimulation of acidic pH and oxidation stress. These foundations indicated that the release mechanisms in response to a single acid pH or oxidation stress or their combination all belonged to the anomalous transport process, wherein the promoted burst drug release for these cases was mainly attributed to the skeleton erosion relative to the diffusion process, owing to the pH-induced shell-shedding and oxidation-induced core-decrosslinking behaviors. Since PEG-based amphiphilic polyurethanes have been proved to possess satisfactory cytocompatibility by many previous researchers [42,43,44,45], this kind of shell-sheddable core-crosslinked micelle could be expected to be a promising nanocarrier for controlled drug delivery.

## 4. Conclusions

In summary, facile designed diselenide-crosslinked shell-sheddable PEGylated polyurethane (PU) micelles with dual pH and oxidation sensitivities were developed. The resulting crosslinked micelles underwent an improved dilution-resistance stability as compared to the non-crosslinked ones, which is beneficial for micellar nanocarriers for maintaining their self-assembly structure during blood circulation. The diselenide-crosslinked micelles possessed a distinct pH-induced PEG shell detachment and oxidation-triggered PU core decrosslinking behaviors, exhibiting an interesting dual pH- and oxidation-triggered degradation behavior. Meanwhile, these crosslinked micelles could efficiently encapsulate hydrophobic IND model drugs with a comparable drug loading content (20.6%) and drug loading efficiency (73.2%). Upon exposure to the acidic pH and/or oxidation circumstances, this kind of micellar nanocarrier displayed an accelerated drug release behavior, demonstrating an enhanced drug bioavailability, which is highly on-demand for nanocarriers to deliver drugs at specific target sites. In light of the excellent performances elaborated above, this kind of shell-sheddable core-crosslinked micelle with a pH and oxidation dual-response could be a potential nanocarrier for on-demand drug delivery.

## Figures and Tables

**Table 1:** The kinetic model parameters obtained from the Ritger and Peppas fitting equation, i.e., kinetic constant (*k*), release exponent (*n*), correlation coefficient (*R^2^*) and the related release mechanism under variable conditions.

Release Medium	*k*	*n*	*R^2^*	Release Mechanism
pH 7.4, 0 mM H_2_O_2_	5.74	0.45	0.93	Pseudo-Fickian
pH 7.4, 50 mM H_2_O_2_	10.71	0.66	0.95	Anomalous transport
pH 6.5, 0 mM H_2_O_2_	10.38	0.62	0.93	Anomalous transport
pH 6.5, 50 mM H_2_O_2_	16.52	0.79	0.93	Anomalous transport
pH 5.0, 0 mM H_2_O_2_	15.08	0.68	0.95	Anomalous transport
pH 5.0, 50 mM H_2_O_2_	20.19	0.79	0.97	Anomalous transport

## Data Availability

The data presented in this study are available on request from the corresponding author.
